# Assessing the relationship between migraine and sino-nasal symptoms and diseases among Syrian Private University students: A case–control study

**DOI:** 10.1097/MD.0000000000041680

**Published:** 2025-02-21

**Authors:** Louloua Al Kadri, Ahmad Nabil Alhouri, Louei Darjazini Nahas

**Affiliations:** a Faculty of Medicine, Syrian Private University, Damascus, Syrian Arab Republic; b Department of Diagnostic Radiology, Damascus University, Damascus, Syrian Arab Republic; c Otorhinolaryngology Department, Syrian Private University, Damascus, Syrian Arab Republic.

**Keywords:** headache, migraine, nasal disease, nasal symptoms, otolaryngology, Syrian Private University

## Abstract

Migraine is a common chronic and disabling condition, diagnosed late in most patients. Furthermore, sino-nasal diseases are severe stressing conditions that can correlate with headaches and migraine. This study aims to assess the relationship between migraine and sino-nasal disorders among Syrian Private University students. A case–control study was conducted among the students of the Syrian Private University in Damascus. Data were obtained using a valid self-administered questionnaire in Arabic to study the prevalence and severity of migraine and sino-nasal diseases using the Migraine Screening Questionnaire and Sino-Nasal Outcome Test 22. The study included 963 students, of whom 417 were cases who had migraines according to the Migraine Screening Questionnaire, and 546 were controls who did not. The Chi-square test assessed the relationship between case–controls and study variables. *P*-value was considered at < .05. Out of 963 students, 296 were male and 667 were female, with an average age of 23.8. Most students were from the Faculty of Medicine (27.1%) and were in their final years of study (24%). Most sino-nasal diseases are related to migraine, including nasal obstruction, the need to blow the nose, ear pain, ear fullness, or pain in facial bones. Sino-nasal outcome test score was significantly related to migraine. The severity of sino-nasal symptoms was significantly associated with migraine. The results of this study indicate that the diseases and symptoms of the nose and sinuses are significantly associated with migraine. Healthcare providers must raise awareness about this relationship for further evaluation and research and for early provision of the appropriate advice and treatment to improve patient’s quality of life and minimize disability.

## 1. Introduction

Migraine is a chronic nerve illness that can occur with or without an aura and is characterized by attacks of excruciating headaches as well as autonomic and neural symptoms.^[1]^ Acute migraines have been classified by the World Health Organization as one of the most disabling chronic diseases, along with quadriplegia, psychosis, and dementia.^[1]^ A study conducted about the prevalence of migraine headaches among students of medical faculties at the University of Aleppo showed that 17.6% of the students suffered from migraine headaches, compared to the international prevalence of migraine at 10%.^[2]^ Chronic rhinosinusitis (CRS) is one of the most prevalent nasal disorders that may resemble migraines in terms of symptoms and demographics.^[3,4]^ Nasal septal deviation plays a critical role in increased nasal obstruction symptoms, the aesthetic appearance of the nose, and increased nasal airflow resistance.^[5]^ Allergic rhinitis is a condition characterized by symptomatic nasal inflammation triggered by exposure to allergens, resulting from an IgE-mediated immune response in the nasal membranes.^[6]^ Nasal polyps are benign, inflammatory, and hyperplastic growths on the mucous membranes of the sinuses. Its most common presentation is in patients with CRS.^[7]^ As they affect up to 4% of the population, nasal polyps are recognized as one of the most common nasal inflammatory masses.^[8]^ They usually exhibit symptoms of postnasal drip, nasal obstruction, anosmia, rhinorrhea, and, less frequently, facial pain.^[8]^ Signs and symptoms of acute and CRS are similar, but the difference is in the duration.^[9]^ Rhinosinusitis must have at least 2 symptoms to be diagnosed. One of them must be (i.e., nasal blockage/obstruction/congestion or rhinorrhea) combined with facial pain or pressure and or anosmia.^[6]^ It is diagnosed as a cause of headaches because it is believed that pain above the sinuses must be associated with them.^[10]^ However, migraines and tension headaches often cause frontal headaches, and there is still debate about whether nasal obstruction will cause chronic headaches.^[10]^ Headaches caused by rhinosinusitis are deep and dull, accompanied by a feeling of fullness and heaviness,^[10]^ and are often continuous and rarely intermittent.^[6]^ Migraine headaches and headaches resulting from sinus disturbances are often mixed up due to their similar pain localizations.^[5]^ Furthermore, it was found that migraine headaches are frequently accompanied by symptoms of cranial autonomic symptoms, such as nasal congestion and rhinorrhea.^[11]^ Some patients suffer from nasal congestion and localized pain in facial bones during a migraine attack, resulting from vasodilation in the turbinates.^[6]^ A study indicates that headaches caused by nasal diseases are often secondary to prolonged mucosal contact points and sinus hypoxia following ischemia or pressure caused by the growth of nasal polyps.^[12]^ Therefore, not all headaches accompanied by nasal symptoms are migraines. Even though headaches can occasionally be attributed to rhinosinusitis, up to 90% of suspected sinus headaches fulfill the diagnostic criteria for migraine.^[13]^ According to Schreiber et al, 88% of 2991 individuals who presented to a general practitioner with a sinus headache were diagnosed with migraines.^[14]^ Sinus headaches can also be caused by comorbidities of both disorders, which is not uncommon given the high prevalence of both rhinosinusitis and migraines.^[15]^

This study aims to assess the relationship between migraine and sino-nasal diseases and symptoms among a sample of students at the Syrian Private University (SPU).

## 2. Methods and materials

### 2.1. Study design and participants

This case–control study was approved by the Institutional Review Board of the SPU; Institutional Review Board 1-14-23. The inclusion criteria included students at the SPU who were between 18 and 50 years old. The exclusion criteria included students under 18 or over 50 years of age, participants who were not students at SPU, students with acute or chronic illnesses, and students undergoing long-term treatments. Using the sample size calculator, a sample size of 357 was calculated based on a 5% margin of error and a 95% confidence factor for a population of 5000 students. Random sampling was used to collect data between January 14, 2023 and March 1, 2023. The study’s objectives were explained to the students in the written form attached to the questionnaire (File S1, Supplemental Digital Content, http://links.lww.com/MD/O426). Informed consent was obtained from the students who filled out the questionnaire. They were told that all their responses were anonymously recorded and that responding to all questions was not mandatory. Data were collected via paper-based surveys in Arabic and entered into Excel by the authors. Using the Migraine Screening Questionnaire (MSQ), the students were classified into 2 groups: where the study included 963 students, of whom 417 had migraine (cases), and 546 did not have migraine (controls). Sino-nasal disease and symptoms were mainly assessed by the validated Arabic version of the Sino-Nasal Outcome Test 2022 (SNOT-22),^[16]^ which studies the presence and severity of different sino-nasal symptoms.

### 2.2. Questionnaire

The questionnaire consists of 3 parts.

#### 2.2.1. Demographic information, social habits, and medical history

This section included questions about age, gender, faculty, academic year, and social habits such as smoking and alcohol consumption. Additionally, it inquires about the presence of acute or chronic disease, the current use of any long-term treatment, and any past diagnosis of migraine by a healthcare professional.

#### 2.2.2. MSQ of 5 items

It is a questionnaire validated using the Cronbach α test with a score of 0.83, which is used to investigate the presence of migraine headaches. It consists of 5 questions concerning headache intensity and duration of episodes, nausea, photophobia, phonophobia during headache attacks, and the impact of headaches on working life. Each question equaled 1 point, and a final score of 4 or more points was considered positive for migraine. The translated Arabic version used by this study’s questionnaire has a sensitivity of 0.95 and specificity of 0.99, according to the study that validates this tool.^[16]^ The questionnaire also included a question about the number of days the participants suffered a headache in the past 2 weeks and a question that rates the headache intensity on a scale of 1 to 10.

#### 2.2.3. The 14-item SNOT-22 results in scale

Participants answered the validated Arabic version of the SNOT-22 scale to determine the severity of nasal and sinus disease and assess nasal, auricular, and facial symptoms. The severity of these diseases is determined by choosing the appropriate answer from these options (no problem, very mild problem, mild problem, moderate problem, severe problem, very severe problem). We also inquired about any past diagnosis by a medical professional of deviated nasal septum, nasal turbinate hypertrophy, allergic rhinitis, chronic or acute sinusitis, or nasal polyposis, in addition to any history of nasal surgery.

### 2.3. Statistical analysis

The data were analyzed using the Statistical Package for Social Sciences version 25.0, showing numbers and percentages (for categorical variables), averages, and standard deviations (for continuous variables). The MSQ was calculated, where each “yes” answer to one of the scale’s 5 items equals 1 point. A score of 4 or more points would diagnose a migraine headache (migraine). The SNOT-22 score was also calculated, whereby each student would rate each symptom from zero to 5, according to the severity of symptoms. The Chi-square independence test was used to test the independence of qualitative variables, and the independent sample *T* test was used to assess the relationship between the SNOT-22 scale and cases and controls. A multiple linear regression analysis was conducted to examine the predictors of migraine severity, using MSQ as the dependent variable. The predictors included the SNOT-22 scale, age (in years), and the presence of chronic or acute sinusitis. The analysis aimed to identify significant predictors of migraine severity while controlling for potential confounding variables. The assumptions of linear regression were tested, including linearity, normality, multicollinearity, and homoscedasticity. The significance level was set at *P* < .05. Regression coefficients, standardized coefficients, t-values, and confidence intervals were reported to interpret the impact of each predictor. A *P*-value < .05 was adopted as statistical significance with a confidence interval of 95%. The effect size is determined by subtracting the mean of the treatment group from the mean of the control group and then dividing this difference by the standard deviation of one of the groups.^[17]^ Effect size is classified into 4 categories: trivial, small, moderate, or large, according to specific numerical thresholds: <0.1, between 0.1 and 0.3, between 0.3 and 0.5, and >0.5, respectively.^[17]^

## 3. Results

### 3.1. Sample characteristics

Of 963 students who met the inclusion criteria, 30.7% were male, 69.3% were female, and 63.6% were younger than 25. Most students were studying at the Faculty of Medicine; 27.1% and 24% were in their senior year. Three hundred fifty-two (36.6%) of the sample declared their smoking habit and 73 (7.6%) alcohol consumption (Table 1).

**Table 1:** Sample demographic information N = 963.

Variable (N = 963)		N (%)
Sex	Male	296 (30.7%)
	Female	667 (69.3%)
Age	25>	612 (63.6%)
	25≥	350 (36.3%)
Faculty	Medicine	261 (27.1%)
	Dentistry	169 (17.5%)
	Pharmacy	155 (16.1%)
	Engineering	228 (23.7%)
	Business Administration	150 (15.6%)
Academic year	1st	98 (10.2%)
	2nd	125 (13%)
	3rd	125 (13%)
	4th	207 (21.5%)
	5th	177 (18.4%)
	6th	231 (24%)
Social habits	Smoking	352 (36.6%)
	Alcohol consumption	73 (7.6%)

### 3.2. Characteristics of migraine headaches in students

Thirty-one point nine percent of the students claimed that a medical professional had previously diagnosed them with migraine. Additionally, 54.9% reported experiencing severe and frequent headaches, and 49.9% reported a headache lasting more than 4 hours. In addition, 13.5% evaluated the severity of their headaches during the past 2 weeks as 5 on a scale of 0 to 10 (Table 2).

**Table 2:** Migraine characteristics in students.

Variable (N = 963)		N (%)
Have you ever been diagnosed with migraine?	Yes	307 (31.9%)
	No	656 (68.1%)
Do you suffer from severe and frequent headaches?	Yes	529 (54.9%)
	No	434 (45.1%)
Do you suffer from headaches that last for more than 4 hours?	Yes	481 (49.9%)
	No	482 (50.1%)
Do you usually suffer from nausea when you have a headache?	Yes	374 (38.8%)
	No	589 (61.2%)
Does light or noise bother you when you have a headache?	Yes	650 (67.5%)
	No	313 (32.5%)
Does a headache limit any of your physical or intellectual activities?	Yes	678 (70.4%)
	No	285 (29.6%)
Number of days with headache in the past 2 weeks	<7 days	846 (87.9%)
	More than 7 days	117 (12.1%)
On a scale of 0 to 10, how severe is the headache	0	108 (11.2%)
	1	23 (2.4%)
	2	45 (4.7%)
	3	64 (6.6%)
	4	99 (10.3%)
	5	130 (13.5%)
	6	117 (12.1%)
	7	155 (16.1%)
	8	118 (12.3%)
	9	44 (4.6%)
	10	60 (6.2%)
Headache severity out of 10	5>	339 (35.2%)
	5≤	624 (64.8%)
Results of Migraine Screening Questionnaire (MSQ)	Migraine	417 (43.3%)
	None	546 (56.7%)

### 3.3. Medical and surgical history of students

A 20.9% of the sample mentioned having a history of nasal surgery, compared to 79.1% who did not. Additionally, 37.3% of them have no history of sino-nasal diseases, and 31.4% of the sample suffer from allergic rhinitis (Table 3).

**Table 3:** Medical and surgical nasal history of students.

Variable (N = 963)		N (%)
History of nasal surgery	Yes	201 (20.9%)
	No	762 (79.1%)
History of sino-nasal diseases	Nasal septum deviation	288 (29.9%)
	Nasal turbinate hypertrophy	197 (20.5%)
	Allergic rhinitis	302 (31.4%)
	Acute or chronic sinusitis	298 (30.9%)
	Nasal polyps	23 (2.4%)
	No history	359 (37.3%)

### 3.4. Possible nasal symptoms in the past 2 weeks

19.5% and 17.8% reported suffering to a mild degree from sneezing and nasal blockage, respectively, compared to 32.2% and 32.4% who did not suffer from that, respectively. The majority reported having the following symptoms to a very mild degree: runny nose 19.4%, loss or decreased sense of smell or taste 11.7%, coughing 18.7%, post postnasal discharge 16.7%, thick nasal discharge 16.6%, epistaxis 6.9%, snoring 11.5%, a sense of ear fullness 22.7%, dizziness 19.6%, ear pain 15.8%, and the presence of pressure or pain in the face 14.3% (Table 4).

**Table 4:** Possible nasal symptoms during the past 2 weeks.

Symptoms	Extremely severe problem	Severe problem	Moderate problem	Mild problem	Very mild problem	No problem
Need to blow nose	36 (3.7%)	67 (7%)	**175 (18.2%**)	147 (15.3%)	166 (17.2%)	372 (38.6%)
Sneezing	16 (1.7%)	55 (5.7%)	153 (15.9%)	**188 (19.5%**)	241 (25%)	310 (32.2%)
Runny nose	15 (1.6%)	45 (4.7%)	114 (11.8%)	146 (15.2%)	**187 (19.4%**)	456 (47.4%)
Nasal blockage	41 (4.3%)	103 (10.7%)	167 (17.3%)	**171 (17.8%**)	169 (17.5%)	312 (32.4%)
decreased sense of smell and taste	16 (1.7%)	31 (3.2%)	66 (6.9%)	65 (6.7%)	**113 (11.7%**)	672 (69.8%)
Coughing	7 (0.7%)	24 (2.5%)	62 (6.4%)	80 (8.3%)	**180 (18.7%**)	610 (63.3%)
Postnasal discharge (sputum)	22 (2.3%)	51 (5.3%)	120 (12.5%)	103 (10.7%)	**161 (16.7%**)	506 (52.5%)
Thick nasal discharge	17 (1.8%)	43 (4.5%)	86 (8.9%)	109 (11.3%)	**160 (16.6%**)	548 (56.9%)
Epistaxis (frequent nose bleed)	5 (0.5%)	12 (1.2%)	28 (2.9%)	61 (6.3%)	**66 (6.9%**)	791 (82.1%)
Snoring	9 (0.9%)	28 (2.9%)	32 (3.3%)	74 (7.7%)	**115 (11.9%**)	705 (73.2%)
Ear fullness	30 (3.1%)	49 (5%)	113 (11.7%)	139 (14.4%)	**219 (22.7%**)	413 (42.9%)
Dizziness	34 (3.5%)	47 (4.9%)	117 (12.1%)	156 (16.2%)	**189 (19.6%**)	420 (43.6%)
Ear pain	23 (2.4%)	30 (3.1%)	81 (8.4%)	99 (10.3%)	**152 (15.8%**)	578 (60%)
Facial pain/pressure	51 (5.3%)	51 (5.3%)	104 (10,8%)	116 (12%)	**138 (14.3%**)	503 (52.2%)

The bold values indicate the highest percentages of sample answers regarding each symptom.

### 3.5. Case–control investigation

The results showed a statistically significant relationship between cases and controls with demographic factors; most students who suffered from migraine headaches were female, 79.9%, compared to 20.1% being males (*P* < .001). Most of those who suffered from migraines were under the age of 25 (57.1%), compared to those aged 25 years or older (42.9%) (*P* < .001) (Table 5).

**Table 5:** The relationship between cases and controls in terms of demographic factors and social habits.

Demographic factors and social habits		Migraine		Chi-square	*P*-value
		Cases incidence (%)	Controls incidence (%)		
Sex	Male	84 (20.1%)	212 (38.8%)	38.768	**<.001**
	Female	333 (79.9%)	334 (61.2%)		
Age	25>	238 (57.1%)	374 (68.6%)	13.615	**<.001**
	25≥	179 (42.9%)	171 (31.4%)		
Faculty	Medicine	68 (16.3%)	193 (35.3%)	46.870	**<.001**
	Dentistry	82 (19.7%)	87 (15.9%)		
	Pharmacy	87 (20.9%)	68 (12.5%)		
	Engineering	107 (25.7%)	121 (22.2%)		
	Business Administration	73 (17.5%)	77 (14.1%)		
Academic year	1	47 (11.3%)	51 (9.3%)	5.160	.4
	2	49 (11.8%)	76 (13.9%)		
	3	52 (12.5%)	73 (13.4%)		
	4	99 (23.7%)	108 (19.8%)		
	5	79 (18.9%)	98 (17.9%)		
	6	91 (21.8%)	140 (25.6%)		
Social habits	Smoking	152 (63.5%)	200 (36.6%)	0.003	.9
	Alcohol consumption	23 (5.5%)	50 (9.2%)	4.476	.3

The bold values indicate significant *P*-values.

The results showed a statistically significant relationship between cases and controls with medical and surgical history: most of the students who had migraines had a history of acute or CRS; significantly, 38.8% compared to the controls, 24.9% (*P* < .001). Most students who did not suffer from migraines had no history of sino-nasal diseases, significantly 40.8% of controls, compared to 32.6% of cases (*P* = .009). Most students with migraine headaches had headaches of intensity greater than or equal to 5 out of 10 (92.8%) compared to the controls (43.4%) (*P* < .001) (Table 6).

**Table 6:** The relationship between cases and controls in terms of medical and surgical history.

Medical and surgical history		Migraine		Chi-square	*P*-value
		Cases incidence (%)	Controls incidence (%)		
History of nasal surgery	Yes	329 (78.9%)	433 (79.3%)	0.024	.9
	No	88 (21.1%)	113 (20.7%)		
History of sino-nasal diseases	Nasal Septum deviation	130 (31.2%)	158 (28.9%)	0.565	.5
	Nasal turbinate hypertrophy	93 (22.2%)	104 (19%)	1.539	.2
	Allergic rhinitis	140 (33.6%)	162 (29.7%)	1.673	.2
	Acute or chronic sinusitis	162 (38.8%)	136 (24.9%)	21.502	**<.001**
	Nasal polyps	10 (2.4%)	13 (2.4%)	0.000	>.99
	No history	136 (32.6%)	223 (40.8%)	6.847	**.009**
Previous migraine diagnosis by a medical professional	No	162 (38.8%)	493 (90.5%)	289.596	**<.001**
	Yes	255 (61.2%)	52 (9.5%)		
Number of days with headache in the past 2 weeks	<7	334 (80.1%)	512 (93.8%)	41.436	**<.001**
	More than 7	83 (19.9%)	34 (6.2%)		
Headache intensity on a scale from 1 to 10	<5	30 (7.2%)	309 (56.6%)	252.936	**<.001**
	≥5	387 (92.8%)	237 (43.4%)		

The bold values indicate significant *P*-values.

The results showed a statistically significant relationship between cases and controls with nasal symptoms: the students with migraine headaches had significantly severe/very severe nasal obstruction, 19.4%, compared to the 11.5% controls. In contrast, the students in the control group did not suffer from nasal obstruction, and 35.5% of the students in the case group did not suffer from nasal obstruction, compared to 28.3% of the students in the case group (*P* = .001). Students who had migraine headaches had severe/very severe ear fullness of 10.1%, significantly compared to controls 6.8%. On the other hand, 49.8% of the students in the control group did not suffer from ear fullness, compared to 33.8% of the students in the case group (*P* < .001). The students in the case group had severe/very severe facial bone pressure or pain at 17.3%, significantly compared to 5.5% of controls. On the other hand, 64.1% of the students in the control group did not suffer from pressure or pain in the facial bones, compared to 36.7% of the students in the case group (*P* < .001). Students with migraine (cases) suffered from severe/extremely severe feelings of imbalance at 12.2% compared to 5.5% of controls. On the other hand, 52.2% of controls denied any feelings of imbalance compared to 32.4% of cases (*P* < .001). Students with migraine (cases) suffered from severe/extremely severe ear pain at 7.4% compared to 4% of controls. On the other hand, 65.9 % of controls denied any ear pain compared to 52.3% of cases (*P* < .001) (Table 7).

**Table 7:** The relationship between cases and controls in terms of SNOT-22 factors.

Sino-nasal symptoms		Migraine		Chi-square	*P*-value
		Cases incidence (%)	Controls incidence (%)		
Need to blow nose	No problem	155 (37.2%)	217 (39.7%)	10.143	**.01**
	Very mild/mild	57 (13.7%)	109 (20%)		
	Moderate	154 (36.9%)	168 (30.8%)		
	Severe/extremely severe	51 (12.2%)	52 (9.5%)		
Sneezing	No problem	146 (35%)	164 (30%)	6.995	.08
	Very mild/mild	88 (21.1%)	153 (28%)		
	Moderate	154 (36.9%)	187 (34.2%)		
	Severe/extremely severe	29 (7%)	42 (7.7%)		
Runny nose	No problem	188 (45.1%)	268 (49.1%)	7.562	.07
	Very mild/mild	73 (17.5%)	114 (20.9%)		
	Moderate	131 (31.4%)	129 (23.9%)		
	Severe/extremely severe	25 (6%)	35 (6.4%)		
Nasal blockage	No problem	118 (28.3%)	194 (35.5%)	16.386	**<.001**
	Very mild/mild	64 (15.3%)	105 (19.2%)		
	Moderate	154 (36.9%)	184 (33.7%)		
	Severe/extremely severe	81 (19.4%)	63 (11.5%)		
Decreased sense of smell/taste	No problem	276 (66.2%)	396 (72.5%)	5.690	.1
	Very mild/mild	52 (12.5%)	61 (11.2%)		
	Moderate	63 (15.1%)	68 (12.5%)		
	Severe/extremely severe	26 (6.2%)	21 (3.8%)		
Coughing	No problem	265 (63.5%)	345 (63.5%)	0.142	>.99
	Very mild/mild	76 (18.2%)	104 (19%)		
	Moderate	62 (14.9%)	80 (14.7%)		
	Severe/extremely severe	14 (3.4%)	17 (3.1%)		
Post nasal discharge	No problem	207 (49.6%)	299 (54.8%)	3.279	.4
	Very mild/mild	70 (16.8%)	91 (16.7%)		
	Moderate	107 (25.7%)	116 (21.2%)		
	Severe/extremely severe	33 (7.9%)	40 (7.3%)		
Thick nasal discharge	No problem	224 (53.7%)	324 (59.3%)	6.828	.08
	Very mild/mild	65 (15.6%)	95 (17.4%)		
	Moderate	99 (23.7%)	96 (17.6%)		
	Severe/extremely severe	29 (7%)	31 (5.7%)		
Epistaxis	No problem	342 (82%)	449 (82.2%)	1.153	.8
	Very mild/mild	26 (6.2%)	40 (7.3%)		
	Moderate	40 (9.6%)	49 (9%)		
	Severe/extremely severe	9 (2.2%)	8 (1.5%)		
Snoring	No problem	302 (72.4%)	403 (73.8%)	3.265	.4
	Very mild/mild	45 (10.8%)	70 (12.8%)		
	Moderate	50 (12%)	56 (10.3%)		
	Severe/extremely severe	20 (4.8%)	17 (3.1%)		
Ear fullness	No problem	141 (33.8%)	272 (49.8%)	30.754	**<.001**
	Very mild/mild	96 (23%)	123 (22.5%)		
	Moderate	138 (33.1%)	114 (20.9%)		
	Severe/extremely severe	42 (10.1%)	37 (6.8%)		
Dizziness	No problem	135 (32.4%)	285 (52.2%)	47.321	**<.001**
	Very mild/mild	83 (19.9%)	106 (19.4%)		
	Moderate	148 (35.5%)	125 (22.9%)		
	Severe/extremely severe	51 (12.2%)	30 (5.5%)		
Ear pain	No problem	218 (52.3%)	360 (65.9%)	19.714	**<.001**
	Very mild/mild	75 (18%)	77 (14.1%)		
	Moderate	93 (22.3%)	87 (15.9%)		
	Severe/extremely severe	31 (7.4%)	22 (4%)		
Facial pain/pressure	No problem	153 (36.7%)	350 (64.1%)	101.705	**<.001**
	Very mild/mild	53 (12.7%)	85 (15.6%)		
	Moderate	139 (33.3%)	81 (14.8%)		
	Severe/extremely severe	72 (17.3%)	30 (5.5%)		

The bold values indicate significant *P*-values.

When studying the relationship between cases and controls regarding the average result of the Sino-Nasal Outcome Test, the results showed statistical significance.

The students with migraine had a significantly greater mean SNOT-22 score of 16.7 than students in the control group, 12.7 (*P* < .001). The students with migraines and a history of nasal surgery had a significantly greater SNOT-22 mean of 16.9 than those in the control group, which had a mean of 13 (*P* = .017). The students with migraines and runny noses had a significantly greater SNOT-22 mean of 21.9 than those in the control group at 18.5 (*P* < .001). The students with migraine and nasal blockage had a significantly greater SNOT-22 mean of 20.4 than those in the control group at 17.2 (*P* < .001). The students with migraine and decreased sense of taste and smell had a significantly greater SNOT-22 mean of 26.2 than those in the control group at 21.4 (*P* < .001).

The students with migraine and sneezing had a significantly greater SNOT-22 mean of 20.2 than those in the control group at 16 (*P* < .001). The students with migraine and coughing had a considerably greater SNOT-22 mean of 23.9 than those in the control group at 18.9 (*P* < .001) (Table 8).

**Table 8:** Assessing the relationship between cases and controls with the sino-nasal outcome test results and its factors.

	Migraine		*T* test	*P*-value	Effect size
	Cases Mean (SD)	Controls Mean (SD)			
(Sino-Nasal Outcome Test score)	16.7 (11.6)	12.7 (10.8)	5.498	<.001	0.36
History of nasal surgery	16.9 (11.8)	13 (10.5)	2.412	.02	0.35
Need to blow nose	20.9 (11.4)	17.4 (10.6)	3.868	<.001	0.32
Sneezing	20.2 (11.5)	16 (10.8)	4.717	<.001	0.38
Runny nose	21.9 (11.3)	18.5 (11.1)	3.417	<.001	0.3
Nasal blockage	20.4 (11.1)	17.2 (10.6)	3.697	<.001	0.3
Decreased sense of smell/taste	26.2 (11.1)	21.4 (11.4)	3.616	<.001	0.43
Coughing	23.9 (12.2)	18.9 (11.4)	3.877	<.001	0.42
Post nasal discharge	22.4 (11.6)	19.4 (11)	2.772	.006	0.27
Thick nasal discharge	23.9 (11.1)	20.6 (11)	3.022	.003	0.3
Epistaxis	26.9 (12.8)	22.4 (12.3)	2.342	.02	0.36
Snoring	23.9 (11.1)	20.6 (11.1)	3.022	.003	0.3
Ear fullness	23.1 (13.1)	21.1 (12.1)	1.246	.2	–
Dizziness	20 (11.7)	18.5 (11.3)	1.491	.1	–
Ear pain	23.1 (12)	20.7 (11.6)	1.977	.05	–
Facial pain/pressure	20.6 (11.7)	20 (11.7)	548	.6	–

SD = standard deviation.

The linear regression analysis was conducted to identify predictors of migraine severity, as measured by the MSQ total score. The model included 3 predictors: age in years, nasal and sinus disease (assessed by SNOT-22 scale), and the presence of chronic or acute sinusitis. The overall model was statistically significant, F (3, 961) = 35.642, *P* < .001, with an R-squared value of 0.100, indicating that approximately 10% of the variance in MSQ can be explained by the predictors. The analysis showed that age was also a significant predictor (β = 0.058, *P* < .001), meaning that each additional year of age is associated with a 0.058 unit increase in MSQ (β = 0.190). The coefficient for “SNOT-22” was significant (β = 0.035, *P* < .001), indicating that for each unit increase in “SNOT-22,” the MSQ increased by 0.035 units. The standardized coefficient (β = 0.216) suggests a moderate effect size. Individuals with chronic or acute sinusitis had a significant increase in MSQ (β = 0.324, *P* = .012), with those affected reporting scores that were 0.324 units higher than those without sinusitis (β = 0.082) (Table 9).

**Table 9:** Linear regression results for predictors of migraine.

Variable	Unstandardized coefficients (β)	Standardized coefficients (β)	t	Sig.	95% confidence interval (lower–upper)
Age (by year)	0.058	0.190	6.183	<0.001	(0.04–0.07)
SNOT-22	0.035	0.216	6.642	<0.001	(0.02–0.04)
Chronic or acute sinusitis	0.324	0.082	2.525	0.01	(0.07–0.57)

*R* = 0.316, R² = 0.100, Adjusted *R*² = 0.097, standard error of the estimate = 1.72975, Durbin–Watson = 1.848.

## 4. Discussion

Migraine is a significant health challenge that affects a great part of society. As it is often encountered in the community and directly impacts the quality of life, the significance of adequate and proper treatment options increases. Migraine treatment will be achievable by a clear understanding of the pathogenesis.^[18]^ This study aimed to assess the relationship between migraine and sino-nasal symptoms and diseases among a sample of students at the SPU. It included 963 students. The case group consisted of the students who had migraine at 43.3% with an SNOT-22 mean of 16.7 and a standard deviation of (±11.6), and the control group consisted of the students who did not suffer from migraine at 56.7% with an SNOT-22 mean of 12.7 with a standard deviation of (±10.8). It is worth mentioning that 61.2% of the cases claim to have been previously diagnosed with migraine by a medical professional, as opposed to 38.8% of the cases who denied any previous migraine diagnosis; this emphasizes that migraine is an underdiagnosed disease. The majority of cases were females, at 79.9%. The controls showed minimal variance between the sexes, at 38.8% males and 61.2% females. Additionally, 79.1% of the students denied any history of nasal surgery, and 37.3% denied any history of previous sino-nasal disease.

A study revealed that migraine cases occur most commonly in females.^[19,20]^ This agrees with our study, which showed that most of the students who suffered from migraines were females, 79.9%, compared to 20.1% of males.

It was also shown in the same study that the majority of migraine cases occur at ages 30 to 60 years.^[19,20]^ This is contrary to our study in which most of those who suffered from migraine were <25 years old, 57.1%. Compared to 42.9% of those whose age is greater than or equal to 25 years; we can justify that because our sample is college students.

A study conducted in Poland showed that 90% of headaches associated with rhinosinusitis turned out to be migraines upon proper diagnosis,^[15]^ which agrees with the results of this study, where most of the students who suffered from migraines had a history of acute or CRS, significantly 38.8% compared to the controls 24.9%.

In a study conducted in Turkey on 214 migraine patients, of whom 116 (54.2%) were previously diagnosed with rhinosinusitis, 25% of them had an additional symptom.^[21]^ Additionally, another study in Saudi showed that 37% of migraine headache patients had rhinosinusitis compared to 9.9% of otherwise healthy adults (*P* = .001).^[22]^ Our findings in this study manifest that most students with migraines had a history of acute or CRS; significantly, 38.8% compared to the controls, 24.9% (*P* < .001).

As previously stated, migraine headaches are frequently accompanied by cranial autonomic symptoms, which can include nasal congestion and a runny nose.^[11]^ On the other hand, nasal congestion and rhinorrhea are one of the key symptoms used in diagnosing rhinosinusitis.^[8]^ Therefore, migraines are often misdiagnosed as headaches caused by rhinosinusitis, “sinus headache.”^[23]^

In a study conducted in Germany, it was found that facial pain was found in 2.3% of patients with migraines^[24]^; this agrees with our study with a higher percentage in our study, where the students who suffered from migraine had severe/very severe facial bone pressure or pain at 17.3%, significantly compared to 5.5% controls. In contrast, the students who did not suffer from pressure or pain in the facial bones were in the control group at 64.1%, compared to 36.7% in the case group.

A study showed that 64 out of 90 patients who suffer from ear pain fulfilled the diagnostic criteria for migraine headaches of the International Headache Society.^[25]^ This is consistent with our study, where 47.7% of the students with migraine headaches suffered from earache, compared to 34% of controls. On the other hand, 65.9% of the students in the control group did not suffer from ear pain compared to 52.3% of the students in the case group.

In a study conducted in Georgia, the median of the SNOT-22 scale for people suffering from primary headache syndrome was more than for those suffering from CRS without primary headache syndrome.^[26]^ Two additional studies suggest that subjects who met the diagnostic criteria for migraine and tension-type headache scored higher points on the Sino-Nasal Outcome Test.^[23]^ It agrees with our study, where students who suffered from migraine had a SNOT-22 mean significantly higher at 16.7 compared to students in the control group at 12.7.

A study in Korea showed that nasal septal deviation was accompanied by an increased risk of migraine compared to patients who did not suffer from nasal septal deviation.^[27]^ This is consistent with our study, where the students who had migraine headaches had severe/very severe nasal obstruction at 19.4%, significantly compared to the controls at 11.5%. In contrast, students in the control group did not suffer from nasal obstruction, and 35.5% of the students in the case group did not suffer from nasal obstruction, compared to 28.3% of the students in the control group.

For feelings of disrupted balance, a Brazilian study showed 37% of patients who suffer from migraine without aura had postural symptoms compared to 13% of controls who had postural symptoms,^[28]^ which agrees with our study, where patients who had severe/very severe imbalance feelings were 12.2% in the case group and 5.5% in the control group.

### 4.1. Limitations

There is difficulty in providing the necessary tools to assess the symptoms and nasal diseases among students objectively. The results of this study are limited to a sample of students from 1 university only, which may only be representative of some university students in Syria. The design of the case report study is subject to recall and bias and does not allow conclusions to be drawn about causation. Furthermore, patient classification using MSQ does not equal specialist assessment; apart from this, imaging tests with specialist evaluation to diagnose sinusitis are more precise than SNOT-22; therefore, future studies should be conducted using objective diagnostic tools.

## 5. Conclusion

There is a significant relationship between the diseases and symptoms of the nose and sinuses and migraines. These findings suggest that nasal symptoms and sinus conditions should be considered when assessing and managing migraine patients. According to the study, the mean SNOT-22 score is significantly higher in the cases group than in the control group. Furthermore, conducting a study to evaluate nasal symptoms on a sample of migraine patients who attend the neurologist’s clinic may help further assess the relationship between the 2 diseases.

## Author contributions

**Conceptualization:** Louloua Al Kadri.

**Data curation:** Louloua Al Kadri.

**Formal analysis:** Ahmad Nabil Alhouri.

**Investigation:** Louloua Al Kadri, Ahmad Nabil Alhouri.

**Methodology:** Louloua Al Kadri, Ahmad Nabil Alhouri.

**Project administration:** Louloua Al Kadri.

**Software:** Ahmad Nabil Alhouri.

**Supervision:** Louei Darjazini Nahas.

**Validation:** Louloua Al Kadri, Ahmad Nabil Alhouri.

**Visualization:** Louloua Al Kadri.

**Writing – original draft:** Louloua Al Kadri, Ahmad Nabil Alhouri.

**Writing – review & editing:** Louloua Al Kadri, Ahmad Nabil Alhouri.

## Supplementary Material


