# Supplementary material for: Assessing the relationship between migraine and sino-nasal symptoms and diseases among Syrian Private University students: A case–control study
Source: Medicine (Baltimore). 2025 Feb 21;104(8):e41680. doi: 10.1097/MD.0000000000041680 (PMC11856896; doi:10.1097/MD.0000000000041680)
Supplement: Supplementary file 1 [file medi-104-e41680-s001.docx]

**Supplementary File 1**

**Demographic information:**

1. Are you a student at Syrian Private University?

Yes __ No __

1. Sex:

Male__ Female __

1. Age in years: ___
2. Faculty:

Medicine__ Dentistry __ Pharmacy __ Engineering__ Business Administration __

1. Academic year:

First__ Second__ Third __ Fourth__ Fifth__ Sixth__

1. Habits:

Smoking ___. Alcohol consumption___. Both ___. Neither___.

1. Are you currently diagnosed with any acute or chronic diseases?

Yes__. No__.

1. Are you currently undergoing any long-term treatment for any disease?

Yes__. No__.

**Migraine Diagnosis**:

1. Have you been previously diagnosed with migraine headaches by a healthcare professional?

Yes___. No___.

1. Migraine screening questionnaire:

1. Do you have frequent or intense headaches? Yes___ No___

2. Do your headaches usually last more than 4 hours? Yes___ No___

3. Do you usually suffer from nausea when you have a headache? Yes___ No___

4. Does light or noise bother you when you have a headache? Yes___ No___

5. Does a headache limit any of your physical or intellectual activities? Yes___ No___

1. For how many days in the past two weeks have you had a headache? ____
2. On a scale of 1 to 10, what was the intensity of these headaches? (With 1 being very light and 10 being very severe.) ____

**Sino-Nasal symptoms:**

1. Have you been previously diagnosed with any of the following by a medical professional? (You may choose more than one option)

Deviated nasal septum___. Turbinate hypertrophy___. Allergic rhinitis__. Chronic or acute sinusitis____. Nasal polyposis___. None of the above____.

1. Do you have a history of nasal surgery?

Yes__ No__

1. Considering how severe the problem is when you experience it and how frequently it happens, please rate each item below on how ‘bad’ it is by circling the number that corresponds with how you feel using this scale (0 = No problem, 1 = Very mild problem, 2 = Mild or slight problem, 3 = Moderate problem, 4 = Severe problem, 5 = Problem as bad as it can be)

|  | 0 | 1 | 2 | 3 | 4 | 5 |
| --- | --- | --- | --- | --- | --- | --- |
| 1. Need to blow nose |  |  |  |  |  |  |
| 1. Sneezing |  |  |  |  |  |  |
| 1. Runny nose |  |  |  |  |  |  |
| 1. Cough |  |  |  |  |  |  |
| 1. Postnasal discharge (dripping at the back of your nose) |  |  |  |  |  |  |
| 1. Thick nasal discharge |  |  |  |  |  |  |
| 1. Ear fullness |  |  |  |  |  |  |
| 1. Dizziness |  |  |  |  |  |  |
| 1. Ear pain/pressure |  |  |  |  |  |  |
| 1. Facial pain/pressure |  |  |  |  |  |  |
| 1. Nasal blockage |  |  |  |  |  |  |
| 1. Decreased sense of smell/taste |  |  |  |  |  |  |
| 1. Epistaxis |  |  |  |  |  |  |
| 1. Snoring |  |  |  |  |  |  |

Thank you.
